# R1R2 peptide ameliorates pulmonary fibrosis in mice through fibrocyte migration and differentiation

**DOI:** 10.1371/journal.pone.0185811

**Published:** 2017-10-02

**Authors:** Hou-Yu Chiang, Pao-Hsien Chu, Ting-Hein Lee

**Affiliations:** 1 Department of Anatomy, College of Medicine, Chang Gung University, Taoyuan, Taiwan; 2 Graduate Institute of Biomedical Sciences, College of Medicine, Chang Gung University, Taoyuan, Taiwan; 3 Division of Cardiology, Department of Internal Medicine, Chang Gung Memorial Hospital, Taoyuan, Taiwan; 4 College of Medicine, Chang Gung University, Taoyuan, Taiwan; Centre National de la Recherche Scientifique, FRANCE

## Abstract

Circulating fibrocytes play a key role in the pathogenesis of pulmonary fibrosis. Fibrocytes are bone marrow-derived leukocytes, which enter the lungs in response to their chemoattractant CXCL12 and differentiate into fibroblasts or myofibroblasts, leading to excess deposition of the collagen-rich extracellular matrix. Matrix metalloproteinase (MMP)-9 and MMP-2, secreted by fibrocytes, degrade the subendothelial basement membrane and promote fibrocyte influx into the lungs. Here, we demonstrate that R1R2, a novel peptide derived from the bacterial adhesin SFS, attenuates pulmonary fibrosis by preventing the differentiation of fibrocytes into myofibroblasts and by reducing the invasion of fibrocytes through basement membrane-like proteins. Moreover, our findings reveal dual regulation of R1R2 on MMP-9 through reduced enzymatic activity on gelatin and increased cleavage of CXCL12. These data suggest that R1R2 has potent anti-fibrotic effects against pulmonary fibrosis.

## Introduction

Pulmonary fibrosis is characterized by an accumulation of fibroblasts and myofibroblasts along with excess deposition of a collagen-rich extracellular matrix (ECM) in the interstitia of the lungs, leading to irreversible damage to the lung architecture and fatal impairment of lung function [1, 2]. The pathogenic mechanisms of pulmonary fibrosis remain unclear; evidence suggests that dysregulated wound-healing processes contribute to the development of the disease. In the pathogenesis of pulmonary fibrosis, the epithelial and endothelial cells of the alveolar-capillary barrier release pro-inflammatory and pro-fibrotic mediators such as cytokines, chemokines, and growth factors after injury. These mediators initiate inflammatory cell adhesion and extravasation across the endothelium and its underlying basement membrane (BM) into the lung parenchyma [3, 4]. Additionally, these mediators trigger fibroblast migration, proliferation, activation and transformation into smooth muscle α-actin (SMAA)-expressing myofibroblasts along with ECM protein secretion [3, 4]. Normally, the tissue repair process is completed with the reabsorption of the accumulated ECM and concurrent reestablishment of the alveolar-capillary barrier [5]; however, in pulmonary fibrosis, dysregulated activation of fibroblast or myofibroblast persists even though inflammatory responses are blocked or absent, leading to aberrant fibrotic reactions [6, 7]. Therefore, traditional anti-inflammatory treatments including corticosteroids and cytotoxic agents have shown limited efficacy in patients with pulmonary fibrosis [8].

ECM-producing fibroblasts and myofibroblasts are the main mediators of fibrosis [9]. Studies have reported multiple potential sources of lung fibroblasts, including resident mesenchymal cells, pulmonary epithelial cells in epithelial–mesenchymal transitions, and fibrocytes [9, 10]. Fibrocytes are bone marrow-derived circulating mesenchymal progenitor cells, simultaneously expressing the common leukocyte marker CD45, hematopoietic stem cell antigen CD34, and ECM proteins such as type I collagen and fibronectin (FN) [11–14]. Under physiological conditions, the bloodstream contains few fibrocytes; however, these cells proliferate rapidly and leave the bloodstream to enter sites of injury upon pathological stimulation [15, 16]. The contribution of fibrocytes to pulmonary fibrosis in mice and humans has been reported by several groups [17–22]. Circulating fibrocytes express the chemokine receptor CXCR4 and can be recruited into the lungs in response to the chemoattractant CXCL12, secreted from fibrotic lungs [19]. The expression levels of CD34, CD45, and CXCR4 on circulating fibrocytes decrease after entering tissues and differentiating into ECM-producing fibroblasts and myofibroblasts [19, 22, 23]. Fibrocytes secrete several MMPs, including MMP-2, MMP-7, MMP-8, and MMP-9 [24]. Studies have reported that the inhibition of MMP-2 and MMP-9 significantly attenuates fibrocyte migration through BM-like proteins in vitro [24]. Therefore, methods that block fibrocyte influx into the lung parenchyma and differentiation into fibroblasts and myofibroblasts have therapeutic potential in reducing collagen accumulation and maintaining endothelial integrity in fibrotic tissue.

A novel peptide, R1R2, was initially known to inhibit the deposition of type I collagen in vitro and in vivo by preventing the binding of FN to collagen [25, 26]. In our previous study, we demonstrated that in addition to reducing type I collagen content, R1R2 decreases CD45^+^ cell adhesion and transendothelial migration in vitro and in injured arteries [26], suggesting that it may regulate fibrocyte homing and trafficking in pulmonary fibrosis. In the present study, we delivered R1R2 into bleomycin-treated mice and examined the effects of R1R2 on ECM deposition, fibrocyte influx and differentiation. Moreover, we investigated the cellular and molecular mechanisms by which R1R2 ameliorates pulmonary fibrosis.

## Materials and methods

### Materials

R1R2 and the scrambled peptide were custom-synthesized by Kelowna International Scientific Inc. (Taipei, Taiwan). The sequence of R1R2 used in this study was GLNGENQKEPEQGERGEAGPPLSGLSGNNQGRPSLPGLNGENQKEPEQGERGEAGPP, and that of the scrambled peptide was LGNEGQKNPEREEGQPAGEGLGLSPQNSGNLPRSGGNGLPQEKENEGPEQPERPAGG. For competitive enzyme-linked immunosorbent assay (ELISA), human FN and rat type I collagen were purchased from Sigma-Aldrich (St. Louis, MO, USA). Mouse CXCL12 (SDF-1 beta, BioLegend, San Diego, CA, USA), human CXCL12 (SDF-1 beta, R&D, Minneapolis, MN, USA), human and mouse active MMP-9 (Abcam, Cambridge, MA, USA) and MMP-2 (R&D, Minneapolis, MN, USA) proteins, and DQ Gelatin^®^ (Invitrogen, Waltham, MA, USA) were used for in vitro assays.

### Ethics statement

All animal experiments were approved by and performed in compliance with the Institutional Animal Care and Use Committee at Chang Gung University (IACUC Approval No: CGU15-100).

### Bleomycin model of pulmonary fibrosis analyzed using hydroxyproline assay

C57BL/6 mice (12–18 weeks old) were used. Bleomycin (1.5–3.0 U/kg Blenoxane) was instilled into the tracheas directly, followed by intratracheal treatment with 20 μM R1R2 or the scrambled peptide every 2 days. After 14 days of treatment, the lungs were removed, homogenized, precipitated with trichloroacetic acid, and baked overnight at 110°C in HCl. The samples were reconstituted with water, and the hydroxyproline content was measured using a colorimetric chloramine T assay. Some lungs were fixed with 10% neutral-buffered formaldehyde and embedded in paraffin, after which 5-μm-thick sections were cut and stained with picrosirius red for fibrosis evaluation.

### Picrosirius red staining

After deparaffinization, the sections were stained using a picrosirius red stain kit (Abcam), according to the manufacturer instructions. Finally, the sections were observed under an Olympus AX-70 light microscope with a polarized filter (Olympus, Melville, NY, USA). Images were processed using Photoshop CS6 (Adobe, San Jose, CA, USA).

### Confocal fluorescence imaging

To locate type I collagen, CXCR4, and CD31 in the R1R2- or scrambled peptide-treated fibrotic lung tissues, paraffin sections were deparaffinized, permeabilized with 0.1% Triton-X 100 in phosphate-buffered saline (PBS-T) for 10 min, blocked with 5% normal goat serum in PBS-T, and incubated with primary antibodies to type I collagen (1:250, EMD Millipore, Darmstadt, Germany), CXCR4 (1:300, GeneTex, Irvine, CA, USA), and CD31(1:1000, Novus, Littleton, CO, USA) overnight at 4°C, followed by Alexa Fluor 488- and Alexa Fluor 594-conjugated secondary antibodies (1:100, Invitrogen) for 1 h at room temperature. Confocal images were captured using a Zeiss LSM 780 confocal scanning microscope. Images were processed on Photoshop CS6.

### Immunohistochemistry

Tissues were fixed with 10% neutral-buffered formalin overnight, embedded in paraffin, and sectioned. After deparaffinization, the sections were quenched with 3% H_2_O_2_ and blocked with 5% normal goat serum in 0.1 M phosphate-buffered saline (PBS). The sections were incubated with antiserum to collagen I (1:1000, EMD Millipore), smooth muscle α-actin (SMAA, 1:1000, Sigma, Sigma-Aldrich) and CXCR4 (1:1000, GeneTex, Irvine, CA, USA) overnight at 4°C. After rinsing in 0.1M PBS, the sections were incubated with biotinylated IgG (1:100) for 1 h, and the avidin-biotin complex (Vector, Burlinghame, CA, USA) for another 1 h. Finally, the sections were stained using a DAB kit (Dako Cytomation, Carpinteria, CA, USA), and then observed under the light microscope (Olympus).

### Morphometric analysis

The lung tissues treated with 20 μM R1R2 or the scrambled peptide were isolated 2 weeks after bleomycin instillation for morphometric analyses. The mice were euthanized with isoflurane overdose and then perfused with normal saline and 10% neutral-buffered formalin. The lung tissues were isolated, fixed with 10% neutral-buffered formalin for 24 h, embedded in paraffin, and cross-sectioned into 5-μm-thick sections. The entire length of the lung was sectioned, and five sections located at 200-μm intervals were examined for each mouse. For quantitative immunohistochemistry of type I collagen and picrosirius red staining analyses, we captured the digital images of the lung sections and blindly measured the positive staining area and total tissue areas by using Image Pro Plus (Media Cybernetics Inc., Silver Spring, MD, USA). The data of six or seven mice from each group were averaged, and the mean ± standard error of the mean (SEM) are presented here. For quantitative evaluation of SMAA and CXCR4, four to seven equal-sized fields of the lung tissues were randomly selected per section, and four sections were analyzed per animal. The sum of the optical densities from the lung sections of six or seven mice from each group were averaged, and the mean ± SEM are presented.

### Fibrocyte isolation through flow cytometry

After 7 days of intratracheal bleomycin instillation, the mice were euthanized and their lungs were removed. The cells were harvested from lung digests by using a previously described technique[27]. In brief, pulmonary circulation was perfused with PBS only in the fibrocyte infiltration study to remove fibrocytes from circulation in the lungs. The lungs were minced to a slurry in 15 mL of a digestion buffer per lung (1 mg/mL collagenase, 30 μg/mL DNase, and 10% fetal calf serum). After incubating for 30 min at 37°C, undigested fragments were further dispersed by drawing the solution up and down through the bore of a 10-mL syringe, and they were centrifuged at 500 × *g* for 10 min. After the supernatant was decanted, each pellet was briefly resuspended in a cold NH_4_Cl red blood cell lysing solution, neutralized by adding a complete medium (Dulbecco's modified Eagle’s medium with 10% fetal calf serum), and centrifuged at 500 *g* for 10 min. The cell pellets were resuspended and passed through nylon mesh filters (100-μm pore size). Total lung cell suspension was spun through a 40% Percoll gradient. Cell counts and viability were determined through trypan blue exclusion counting on a hemocytometer. Fc-blocking purified anti-CD16/32 (BioLegend), anti-CD45-PE (BioLegend), anti-collagen I-FITC (Bioss), and anti-CXCR4-APC (BioLegend) antibodies were used for flow cytometry. Fibrocytes were identified as CD45^+^/collagen I^+^/CXCR4^+^. The fibrocytes were sorted on a BD FACSAria Cell Sorter (BD Biosciences, Franklin Lakes, NJ, USA) and then collected for further study including Western blotting, migration, and zymography analyses.

### Immunoblotting

Cells were homogenized using a cold lysis buffer (50 mM Tris-HCl [pH 7.5], 150 mM NaCl, 1% NP-40, and 5 mM EDTA) supplemented with protease and a phosphatase inhibitor cocktail (Thermo Scientific, Waltham, MA, USA). The cell lysates were cleared through centrifugation at 12000 rpm for 15 min at 4°C and then electrophoresed through 10% SDS-PAGE under reducing conditions and transferred to nitrocellulose membranes. The membranes were incubated with antibodies to CXCR4 (1:1000, GeneTex), smooth muscle α-actin (SMAA, 1:1000, Sigma-Aldrich), or gapdh (1:5000, Sigma-Aldrich) overnight at 4°C, followed by horseradish peroxidase-conjugated secondary antibodies (1:12000, Bio-Rad, Hercules, CA, USA) for 1 h at room temperature. Blots were developed using enhanced chemiluminescence (Thermo Scientific).

### Matrigel invasion and migration assays

Fibrocytes pretreated with 1000 nM R1R2 or scrambled peptide for 24 h were seeded into Matrigel-coated or uncoated 5-μm pore-sized transwells in 24-well plates (Corning, Corning, NY, USA) at 5.0 × 10^3^ cells/transwell. In brief, R1R2 or the scrambled peptide were added to the fibrocytes, followed by 20 ng/mL mouse CXCL12 to the bottom wells in 24-well plates for 6 h at 37°C. After the media was aspirated from the top well and washed with PBS three times, the transwells were transferred into new 24-well plates containing 800 μL of a cell dissociation buffer with calcein-AM, and the cells were incubated for 1 h at 37°C in a CO_2_ incubator to detach the transmigrated cells into the buffer. The transmigrated fibrocytes were quantified by the fluorescence intensity of calcein-AM by using a SpectraMax M5 microplate reader (Molecular Device, Sunnyvale, CA, USA) set at 485 nm (excitation)/535 nm (emission).

### Quantitative real-time PCR

Total RNA from cells was isolated using TRIzol (Invitrogen). Total RNA from each sample was reverse-transcribed with a First Strand cDNA Synthesis Kit (GE Healthcare Life Sciences), according to the manufacturer instructions. Quantitative real-time PCR was performed using different sets of quantitative PCR primers (mouse MMP-9: forward 5′-CCTGTAAATCTGCTGAAACC-3′, reverse 5′-TCTGACCTGAACCATAACG-3′; mouse gapdh: forward 5′-CTTCATTGACCTCAACTACAT-3′, reverse 5′-AGACTCCACGACATACTC-3′) and SensiFAST™ SYBR^®^ No-ROX Kit (Bioline, London, UK) on a CFX96 Real-Time PCR Detection System (Bio-Rad). Quantitative real-time PCR was performed on cells treated with the R1R2 peptide compared with scrambled peptide control treatment. Each quantitative real-time PCR was performed at least three times, and the representative results were noted as a fold change relative to the control by using the standard 2^−ΔΔCt^ method[28], where Ct represents the number of cycles required to reach the threshold for the target gene, subtracted from the number of cycles required to reach the threshold for a control housekeeping gene (in this case, gapdh).

### Gelatin zymography

The concentrated media of the isolated fibrocytes treated with 1000 nM R1R2 or scrambled peptide accompanied by mouse CXCL12 (40 ng/mL) was electrophoresed on 10% polyacrylamide gel with gelatin. After the run, the gels were incubated in a zymogram renaturation buffer for 30 min at room temperature and in a zymogram development buffer overnight at 37°C. After the gels were stained with 0.5% Coomassie blue R-250, gel images were captured using image scanners.

### ELISA

To detect the interaction between R1R2 and FN, 96-well plates were coated with 20 μg/mL R1R2, the scrambled peptide, or BSA at 4°C overnight, and nonspecific binding sites were blocked with 1% BSA. FN, type I collagen (10 μg/mL), or PBS were added to the coated plates and incubated at room temperature for 2 h. The wells were washed. Rabbit anti-FN or anti-type I collagen antibodies (Merck Millipore, Billerica, MA) were added for 90 min at room temperature. The wells were washed and incubated with horseradish peroxidase-conjugated secondary antibodies. After washing, peroxidase activity was quantified using 2,2′-azino-bis-(3-ethylbenthiazoline-6-sulfonic acid). Measurements were performed at 405 nm on the microplate reader. To evaluate the binding between R1R2 and MMPs, biotinylated R1R2 or scrambled peptide (20 μg/mL) was added to 96-well plates coated with catalytic domain or FLPs of MMP-9/MMP-2 or BSA (8 μg/mL). Bound R1R2 and scrambled peptide were quantified at 405 nm following the incubation of antibiotin antibodies.

### MMP activity assay on fluorescent gelatin

To quantify the MMP activity on gelatin, a fluorescence gelatinolytic assay was performed. The recombinant catalytic domains or FLPs of MMP-9 or MMP-2 were mixed with 10 times the molar ratio of R1R2 or the scrambled peptide and added to a 96-well plate. Subsequently, the Dye-quenched (DQ)^TM^ gelatin substrate (Molecular Probes, Eugene, OR, USA) was added at a final concentration of 100 μg/mL. The fluorescent signal was measured hourly for 6 h on the microplate reader, with λ_ex_ at 495 nm and λ_em_ at 515 nm.

### CXCL12 degradation

A total volume of 50 μL, containing 2 μg of human CXCL12, 0.1 μg of active human full-length MMP-9 protein, and 100 μg of R1R2 or scrambled peptide in 1× PBS, was incubated for 3 h at room temperature and then electrophoresed on 18% polyacrylamide gel. Subsequently, the gels were stained with 0.5% Coomassie blue R-250; gel images were captured using image scanners.

### Statistical analysis

For in vivo experiments, the data are shown as the mean ± standard error of the mean (SEM). For in vitro experiments, the data shown are the mean ± standard deviation (SD) of a representative experiment; at least 3 independent experiments were performed. Student’s t-test analysis or one-way analysis of variance (ANOVA) and a post hoc test were conducted for data analysis with GraphPad Prism (GraphPad, San Diego, CA, USA). If the normality test failed, a nonparametric Kruskal–Wallis test, followed by Dunn's post hoc test, was used. Results with *P* > 0.05 were considered nonsignificant.

## Results

### R1R2 reduces pulmonary fibrosis severity in bleomycin-treated mice

**Fig 1:**
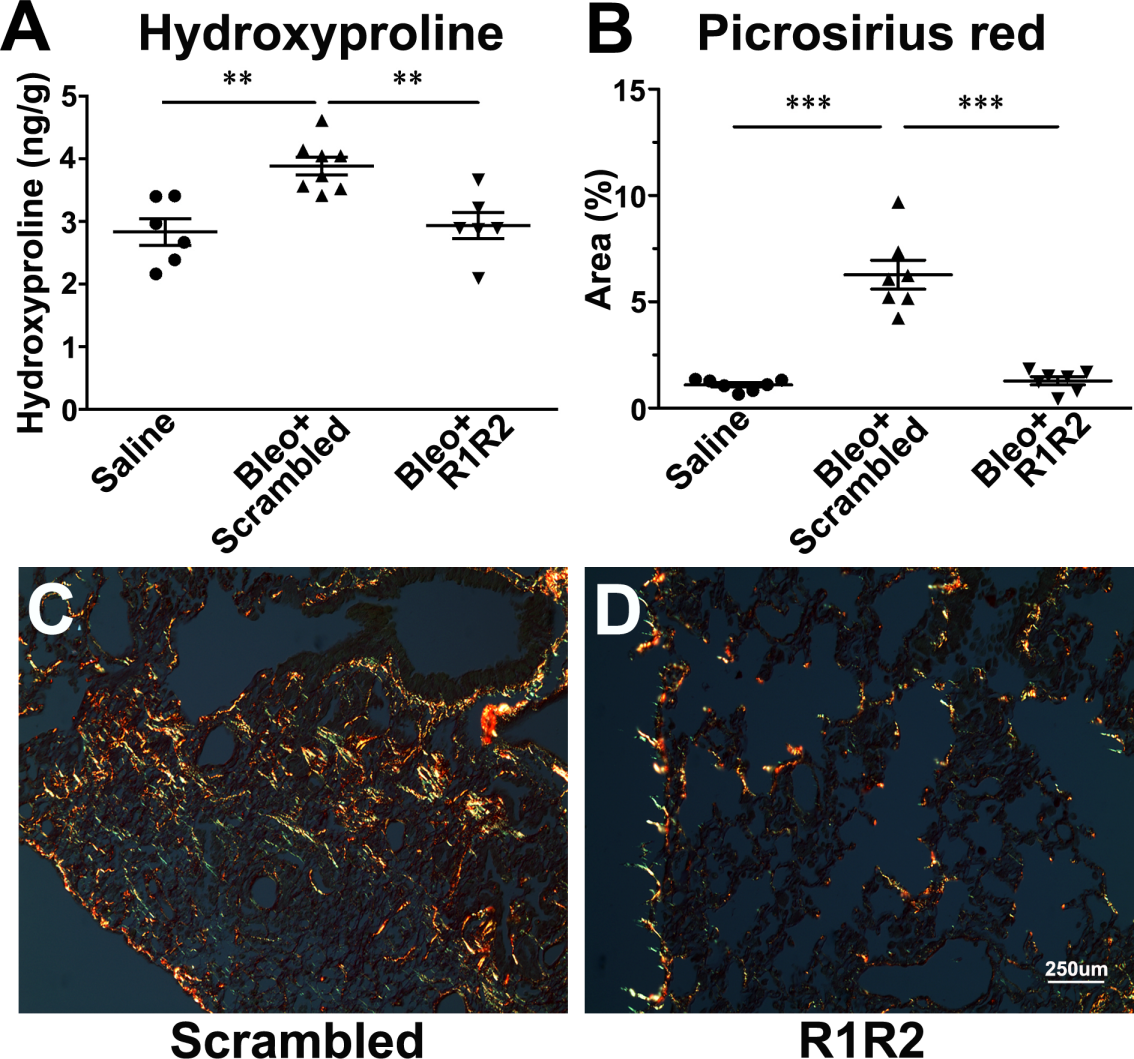
R1R2 reduces collagen content in bleomycin-induced fibrotic lung tissues. (A) Collagen content was biochemically measured using a hydroxyproline assay in the lung 14 days after bleomycin instillation, accompanied by 20 μM R1R2 and the scrambled peptide (Saline: n = 6, Bleo+Scrambled: n = 8, Bleo+R1R2: n = 6). Data are expressed as the mean ± standard error of the mean (SEM). ** *P* < 0.01, one-way analysis of variance (ANOVA) followed by a Bonferroni *t* test for subsequent pairwise comparison. (B–D) Picrosirius red staining of lung sections from the scrambled peptide or R1R2. Quantification of the percentage of the area (B), which is yellow/orange/green (Saline: n = 7, Bleo+Scrambled: n = 7, Bleo+R1R2: n = 7). Data are expressed as the mean ± standard error of the mean (SEM). *** *P* < 0.001, one-way ANOVA followed by a Bonferroni *t* test. Representative images of picrosirius red staining in the fibrotic lungs of the scrambled group (C) and R1R2 group (D). Bar, 250 μm.

**Fig 2:**
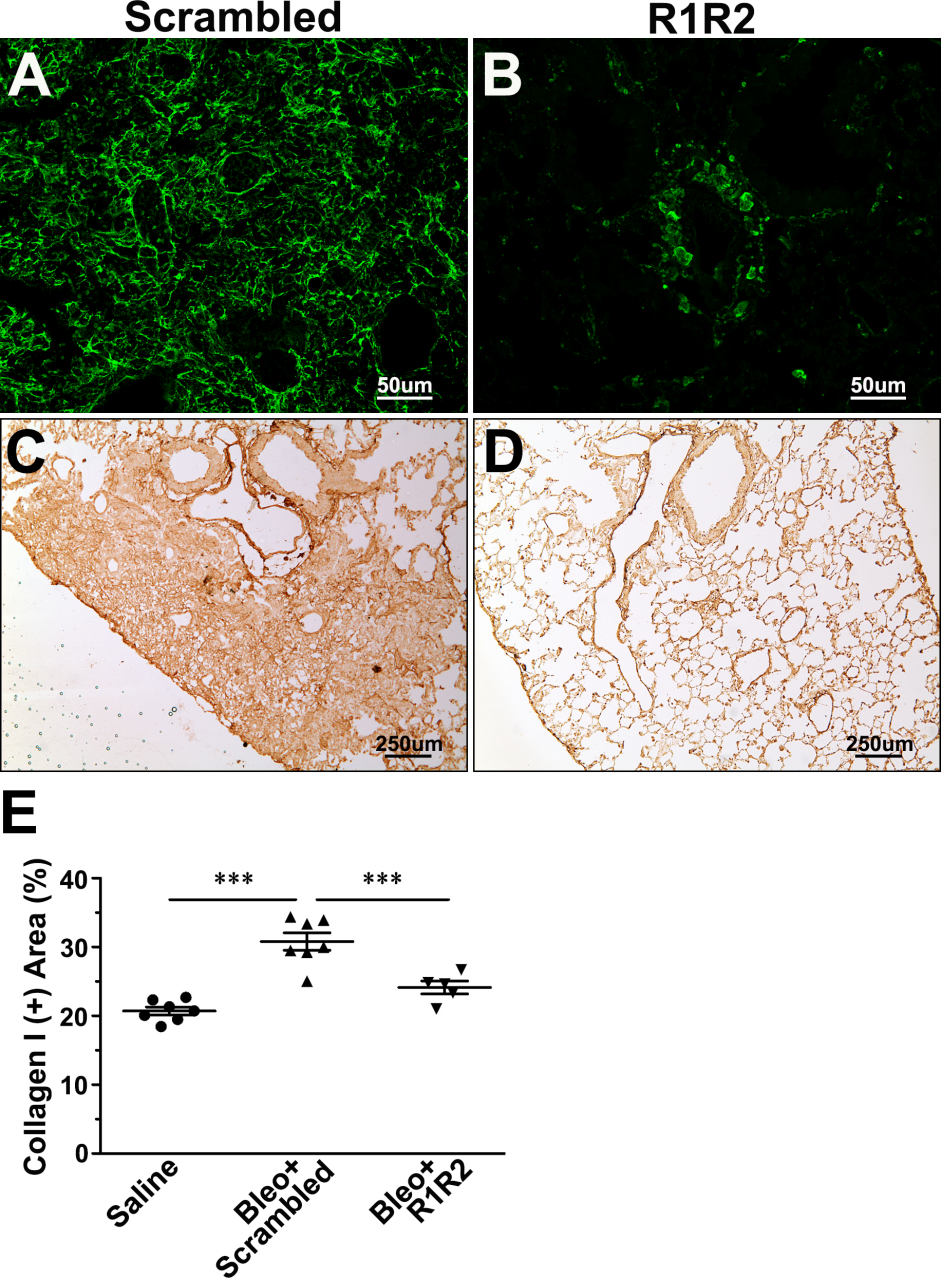
R1R2 reduces collagen type I content in the fibrotic lung tissues. (A, B) Confocal images of immunofluorescence staining for type I collagen 14 days after bleomycin administration, accompanied by R1R2 and the scrambled peptide (20 μM). Bar, 50 μm. (C, D) Representative immunoperoxidase images showing in a bleomycin-treated lung 14 days after bleomycin instillation, accompanied by R1R2 and the scrambled peptide administration (20 μM). (E) Quantification of the percentage of type I collagen^+^ area in a fibrotic lung 14 days after bleomycin instillation (Saline: n = 7, Bleo+Scrambled: n = 7, Bleo+R1R2: n = 5). Data are expressed as the mean ± SEM. *** *P* < 0.001, one-way analysis of variance followed by a Bonferroni *t* test.

Our previous study showed that R1R2 significantly blocks type I collagen deposition and CD45^+^ cell infiltration in a mouse model of vascular remodeling [26]. To assess whether R1R2 reduces the severity of pulmonary fibrosis, we administered R1R2 into bleomycin-treated mice. To optimize bleomycin dose for inducing lung injury, the mice were challenged with various doses of bleomycin intratracheally and then a hydroxyproline assay was performed to evaluate collagen deposition—the main characteristic of pulmonary fibrosis. A dose of 3.0 U/kg induced a significant increase in total lung hydroxyproline content 14 days after bleomycin treatment (S1 Fig). Therefore, we selected 3.0 U/kg to induce pulmonary fibrosis in subsequent experiments. We intratracheally delivered R1R2 or a scrambled peptide into the mice every other day after bleomycin administration. To examine whether the fibrotic response in the bleomycin-treated mice was ameliorated by R1R2, we evaluated collagen levels 2 weeks after bleomycin administration biochemically and histologically. The hydroxyproline assay showed that R1R2 caused a larger reduction in collagen content in the fibrotic lungs than did the scrambled peptide (Fig 1A; 2.94 ± 0.21 [n = 6] vs. 3.88 ± 0.14 [n = 8]). Collagen accumulation was also evaluated using picrosirius red staining on paraffin sections of the lungs (Fig 1B–1D). Under a polarized light microscope, strong yellow-red birefringence indicates type I collagen, whereas weak green birefringence indicates type III collagen [29]. In the present study, quantitative analysis of the picrosirius red-stained slides revealed that the percentage of the collagen^+^ area to the total lung area was reduced to a greater extent in the R1R2-treated lungs compared with that in the scrambled peptide-treated lungs (Fig 1B; 1.28 ± 0.19 [n = 7] vs. 6.274 ± 0.68 [n = 7]). Because type I collagen is the main component of fibrotic tissues [30], we next specifically located type I collagen in the fibrotic lungs through immunohistochemistry (Fig 2). The immunofluorescence images revealed that R1R2 treatment apparently inhibited type I collagen deposition in the lung parenchyma (Fig 2A and 2B). To further quantitate the content of type I collagen, we performed immunoperoxidase staining for type I collagen followed by morphometric analysis (Fig 2C–2E). The results showed consistent reduction in the percentage of the type I collagen^+^ area relative to the total area in the R1R2-treated mice compared with that in the scrambled peptide-treated mice (Fig 2E; 24.12 ± 0.94 [n = 5] vs. 30.77 ± 1.26 [n = 7]).

**Fig 3:**
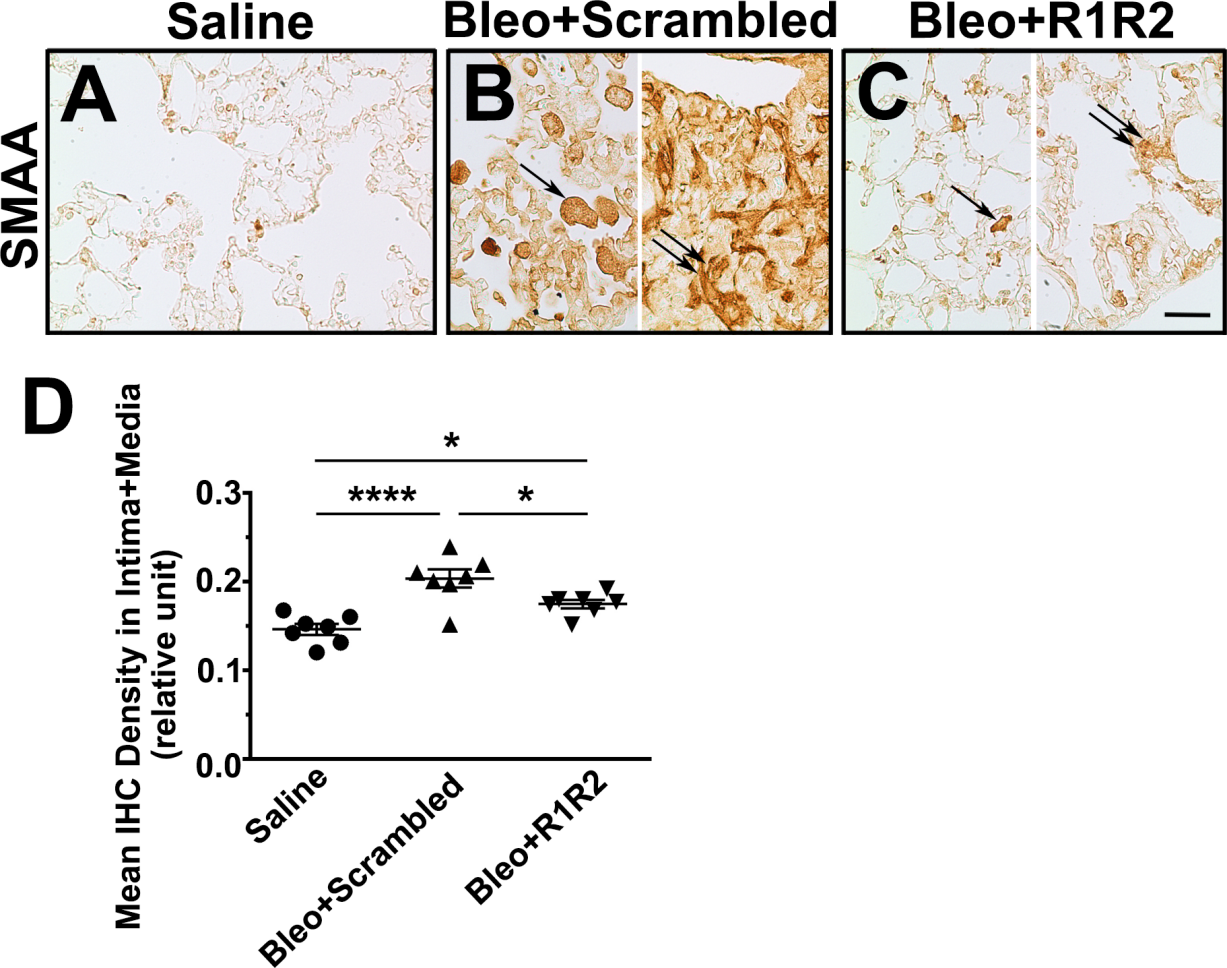
R1R2 attenuates the presence of myofibroblasts. (A)-(C) Representative immunostaining images showing smooth muscle α-actin positive (SMAA^+^) myofibroblasts in a normal or bleomycin-treated lung 14 days after bleomycin instillation, accompanied by R1R2 and the scrambled peptide administration (20 μM). Bar, 30 μm. Arrows and double arrows indicate SMAA^+^ myofibroblasts. (D) Quantitative analysis of immunostaining intensities of SMAA in a fibrotic lung 14 days after bleomycin instillation (n = 7). Data are expressed as the mean ± SEM. * *P* < 0.05 and ****P < 0.0001, one-way analysis of variance followed by a Bonferroni *t* test.

Contractile myofibroblasts have been shown to be the major source of collagen type I gene expression, which are associated with the period of active fibrosis in the lungs [31]. To evaluate the presence of myofibroblasts, we examined the expression of SMAA, a marker for myofibroblasts, through immunohistochemistry in lung sections of the bleomycin-treated mice for 14 days (Fig 3). In the fibrotic lungs of mice treated with the scrambled peptide, SMAA^+^ cells were distributed abundantly (Fig 3B, indicated by an arrow). In some heavily fibrotic area (the right part of Fig 3B), SMAA^+^ cells acquired spindle-cell or stellate-cell morphology, which is the characteristic of reactive myofibroblasts [32] (Fig 3B, indicated by a double arrow). By contrast, in the lungs of mice treated with R1R2, the expression levels of SMAA (Fig 3C, indicated by an arrow) were significantly less than that in the scrambled peptide-treated lungs (Fig 3D; 0.17 ± 0.004 [n = 7] vs. 0.20 ± 0.027 [n = 7]), and only few cells acquired stellate-cell shape (Fig 3C, indicated by a double arrow). Taken together, our results showed that R1R2 significantly reduced the severity of pulmonary fibrosis in a mouse model treated with intrapulmonary bleomycin in terms of attenuated collagen deposition and the presence of myofibroblasts.

### R1R2 may reduce fibrocyte infiltration into fibrotic lungs and prevent myofibroblast differentiation

**Fig 4:**
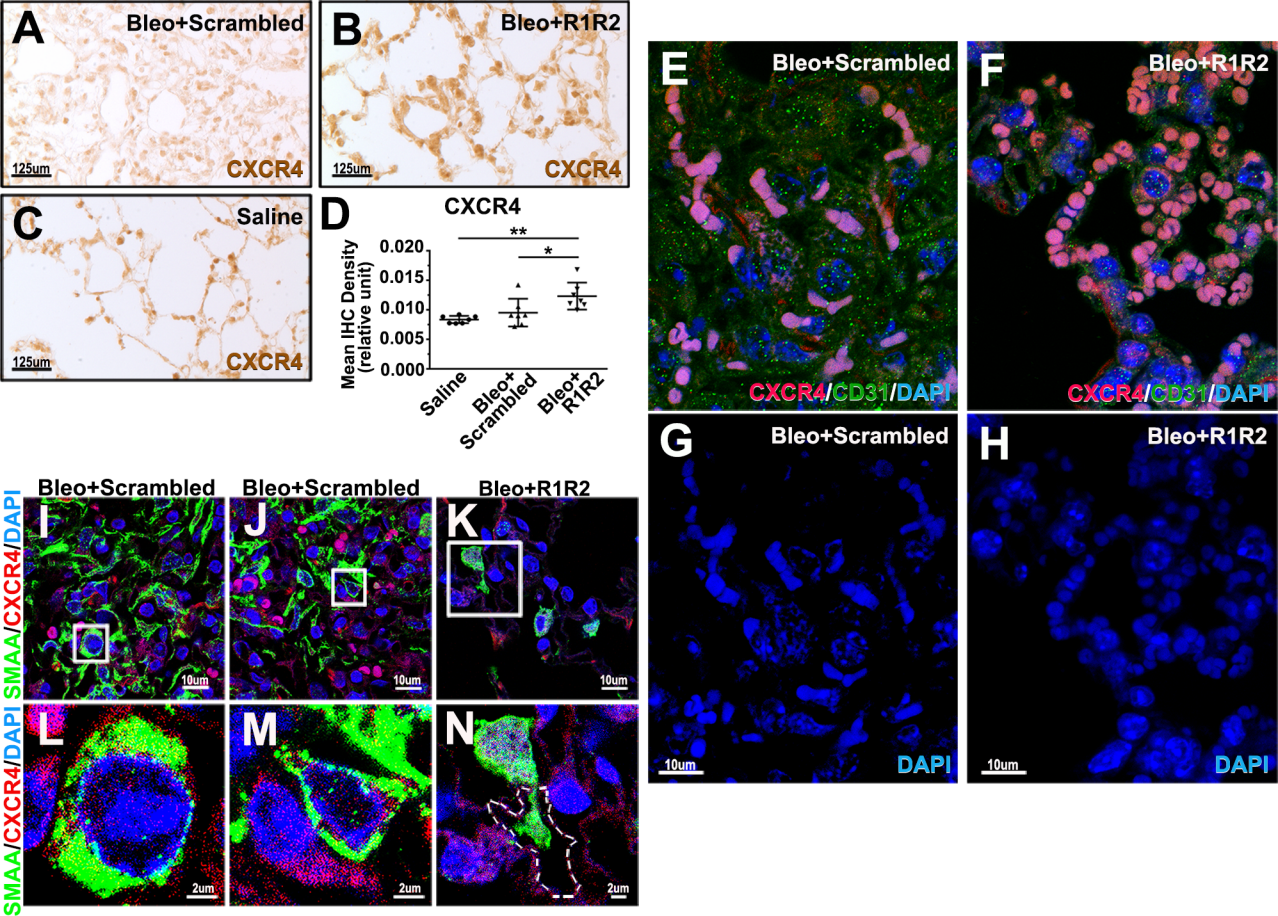
R1R2 decreases CXCR4^+^ cell accumulation in the bleomycin-induced fibrotic lung tissues. (A)-(C) Immunohistochemistry (IHC) for CXCR4 in a normal or bleomycin-treated lung 14 days after bleomycin instillation, accompanied by R1R2 and the scrambled peptide administration (20 μM). (D) Quantitative analysis of immunostaining intensities of CXCR4 in a fibrotic lung 14 days after bleomycin instillation (n = 7). Data are expressed as the mean ± SEM. *P < 0.05 and **P < 0.01, one-way analysis of variance followed by a Bonferroni *t* test. (E)-(H) Confocal images of immunofluorescence staining for CXCR4 (red) and CD31 (green) in a fibrotic lung 14 days after bleomycin instillation, accompanied by R1R2 and the scrambled peptide (20 μM). Sections were counterstained with DAPI. (I)-(N) Confocal images of immunofluorescence staining for CXCR4 (red) and SMAA (green) in a bleomycin-treated lung 14 days after bleomycin treatment. The boxed portion of the low magnification images (I)-(K) is presented at higher magnification (L)-(N) below. A capillary is delineated by dashed lines. Sections were counterstained with DAPI.

Fibrocytes are known to differentiate into SMAA-expressing myofibroblasts after infiltrating into the fibrotic lungs [33, 34]. The present study demonstrates that R1R2 significantly decreases the expression of SMAA in the fibrotic lungs (Fig 3), suggesting that the attenuation of SMAA^+^ myofibroblasts in R1R2-treated lungs might be attributed to the decreased infiltration and differentiation of fibrocytes. CXCR4-expressing bone marrow-derived fibrocytes are recruited into the lung in response to CXCL12 after injury [19, 35]. Because the expression level of CXCR4 on circulating fibrocytes is decreased after entering tissues and differentiating into ECM-producing fibroblasts and myofibroblasts [19, 22, 23], immunohistochemistry for CXCR4 in lung sections of the bleomycin-treated mice for 14 days was performed (Fig 4A–4H). In the fibrotic lungs treated with scrambled peptide, the CXCR4-expressing cells were scattered abundantly in the lung (Fig 4A). By contrast, in R1R2-treated animals, fewer CXCR4^+^ cells were found in the lung (Fig 4B). Interestingly, quantitative immunohistochemistry analysis showed a significant increase of CXCR4 intensity in R1R2-treated lungs compared to that in the scrambled peptide group on 14 day after bleomycin treatment (Fig 4D; 12.32 ± 0.87 x 10^−3^ [n = 7] vs. 9.51 ± 0.89 x 10^−3^ [n = 7]). We next performed immunofluorescence staining for CXCR4 and CD31, an endothelial cell marker, in lung sections of the bleomycin-treated mice for 14 days to locate CXCR4^+^ cells and capillaries at high magnification (Fig 4E–4H). In the scrambled peptide-treated lungs, the CXCR4-expressing cells with faint immunoreactivity were distributed diffusely in the lung interstitia and alveoli (Fig 4E). Notably, CXCR4^+^ cells with strong immunoreactivity in the R1R2-treated lungs were restrained to within the capillaries (Fig 4F). To determine whether these CXCR4^+^ cells are able to differentiate directly into myofibroblasts, immunofluorescence of CXCR4 and SMAA was performed in lung sections of the bleomycin-treated mice for 14 days (Fig 4I–4N). At low magnification, R1R2 treatment obviously decreased the expression of CXCR4 and SMAA compared with that in the lungs treated with the scrambled peptide (Fig 4I–4K). At high magnification, many CXCR4 ^+^ cells co-expressed SMAA in the scrambled peptide-treated lungs, indicating that these CXCR4-expressing cells were directly differentiating into myofibroblasts (Fig 4I, 4J, 4L and 4M). By contrast, only few cells are double positive for CXCR4 and SMAA in R1R2-treated lungs (Fig 4K and 4N). Therefore, we infer from these data that R1R2 prevents myofibroblast differentiation.

**Fig 5:**
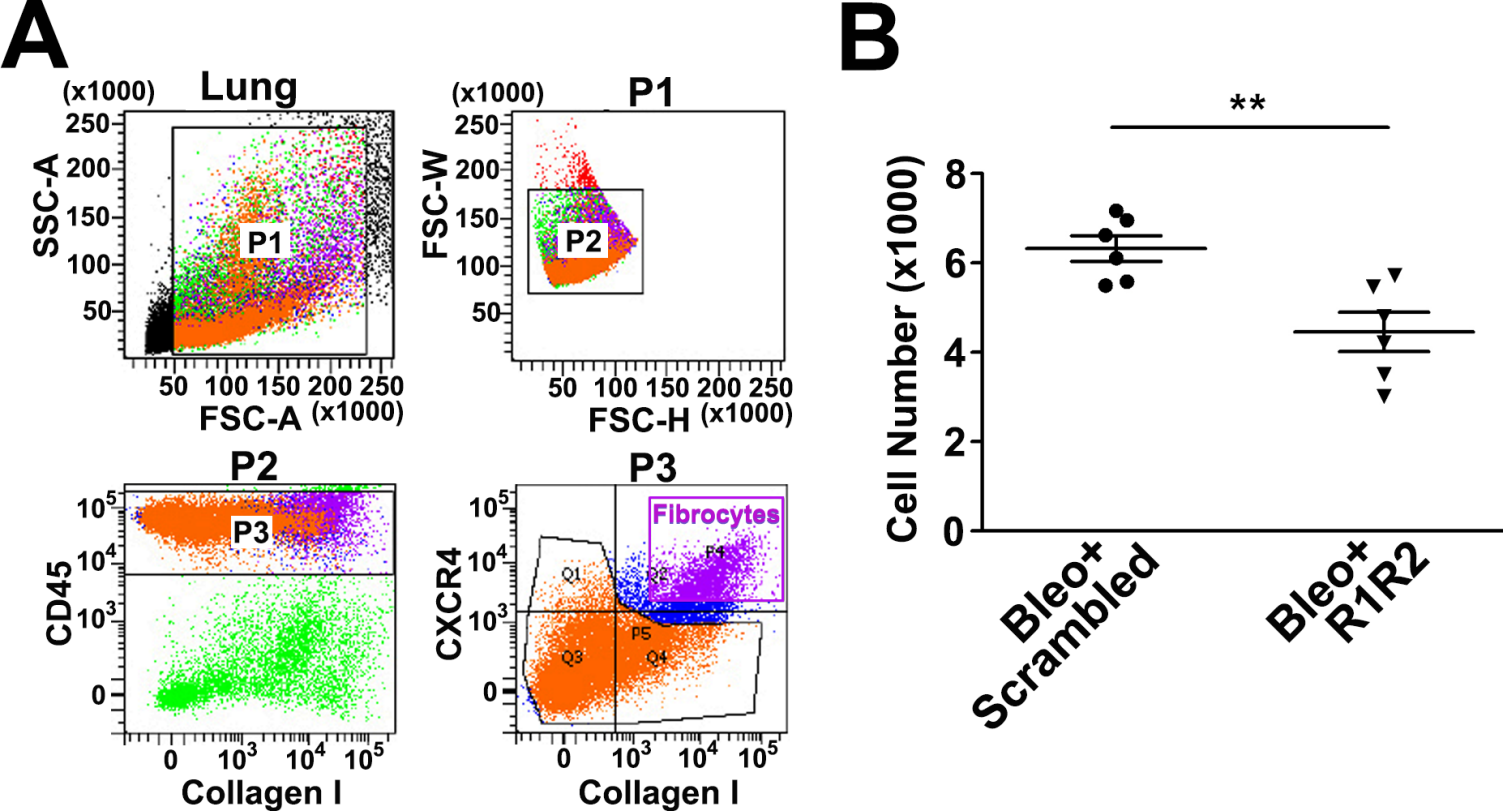
R1R2 impedes fibrocyte infiltration into the bleomycin-induced fibrotic lung tissues. (A) Fibrocytes were isolated and sorted by flow cytometry after staining for CD45, type I collagen (collagen I), and CXCR4. Fibrocytes were identified as CD45^+^, type I collagen^+^, and CXCR4^+^. (B) Cell numbers of CD45^+^/collagen I^+^/CXCR4^+^ fibrocytes are presented through flow cytometry analysis after R1R2 and scrambled peptide treatment in the bleomycin-induced fibrotic lung tissues (n = 6). Data are expressed as the mean ± SEM. **P < 0.01 by *t* test.

To further ascertain that R1R2 decreases the influx of fibrocytes into the lungs in the bleomycin-treated mice, mice were perfused with phosphate-buffered saline (PBS) to wash out all the cells in blood, and the number of fibrocytes—defined as CD45^+^, type I collagen^+^, and CXCR4^+^ cells—entered the lung parenchyma was examined through cell staining and flow cytometry analysis of lung digests on Day 14 of bleomycin treatment with R1R2 or scrambled peptide administration. The number of infiltrating fibrocytes was reduced to 4455 ± 439.6 (n = 6) after R1R2 administration, whereas this number was 6322 ± 287.8 (n = 6) in the scrambled peptide-treated fibrotic lungs (Fig 5), indicating that R1R2 administration blocked the transmigration or the influx of fibrocytes into the lung parenchyma in a mouse model of lung fibrosis.

**Fig 6:**
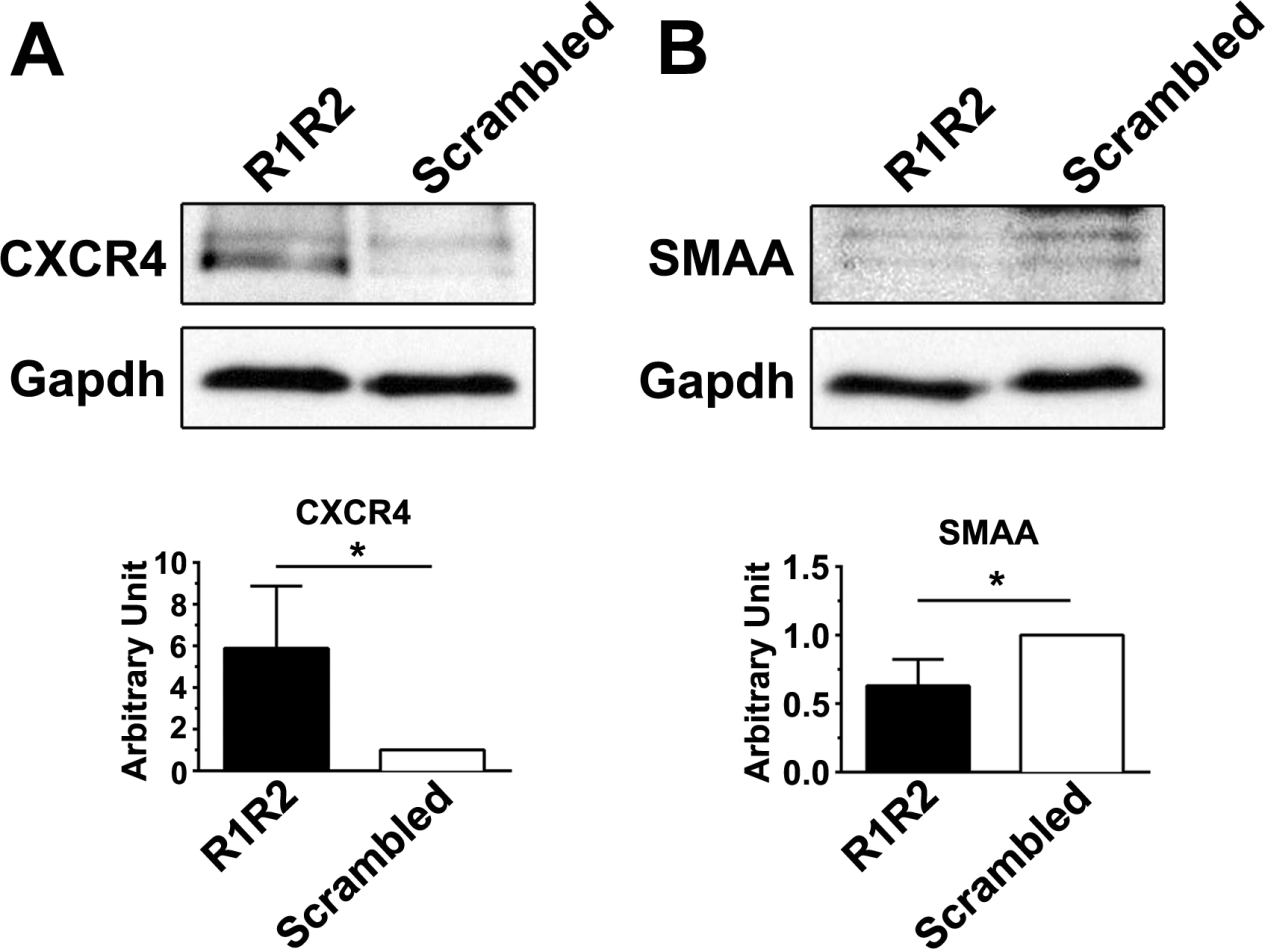
R1R2 regulates fibrocyte differentiation into myofibrblasts. (A, B) Western blot analyses of CXCR4 and SMAA expression in the isolated fibrocytes treated with R1R2 or the scrambled peptide for 24 hours (h). Data are expressed as the mean ± standard deviation (SD). *P < 0.05 by *t* test.

To characterize the effect of R1R2 on fibrocyte differentiation, CD45^+^, type I collagen^+^, and CXCR4^+^ cells were isolated from the lung digest of the bleomycin-treated mice in a cell sorter and then cultured for 48 hours (h) followed by the treatment of R1R2 or the scrambled peptide for 24 h. Consistent with the immunohistochemistry results in Fig 4, R1R2 treatment significantly decreased the protein level of SMAA (Fig 6B, 0.63 ± 0.19 [n = 3] vs. 1 [n = 3]). Simultaneously, Western blot analysis revealed that R1R2 caused a larger increase in CXCR4 expression in fibrocytes compared with the scrambled peptide (Fig 6A, 5.88 ± 2.99 [n = 3] vs. 1 [n = 3]). Thus, in addition to blocking the influx of fibrocytes into the lung parenchyma in vivo, we have proven that R1R2 administration also prevents fibrocytes from differentiating into myofibroblasts.

### R1R2 regulates the proteolytic activity of MMP-9 to BM

**Fig 7:**
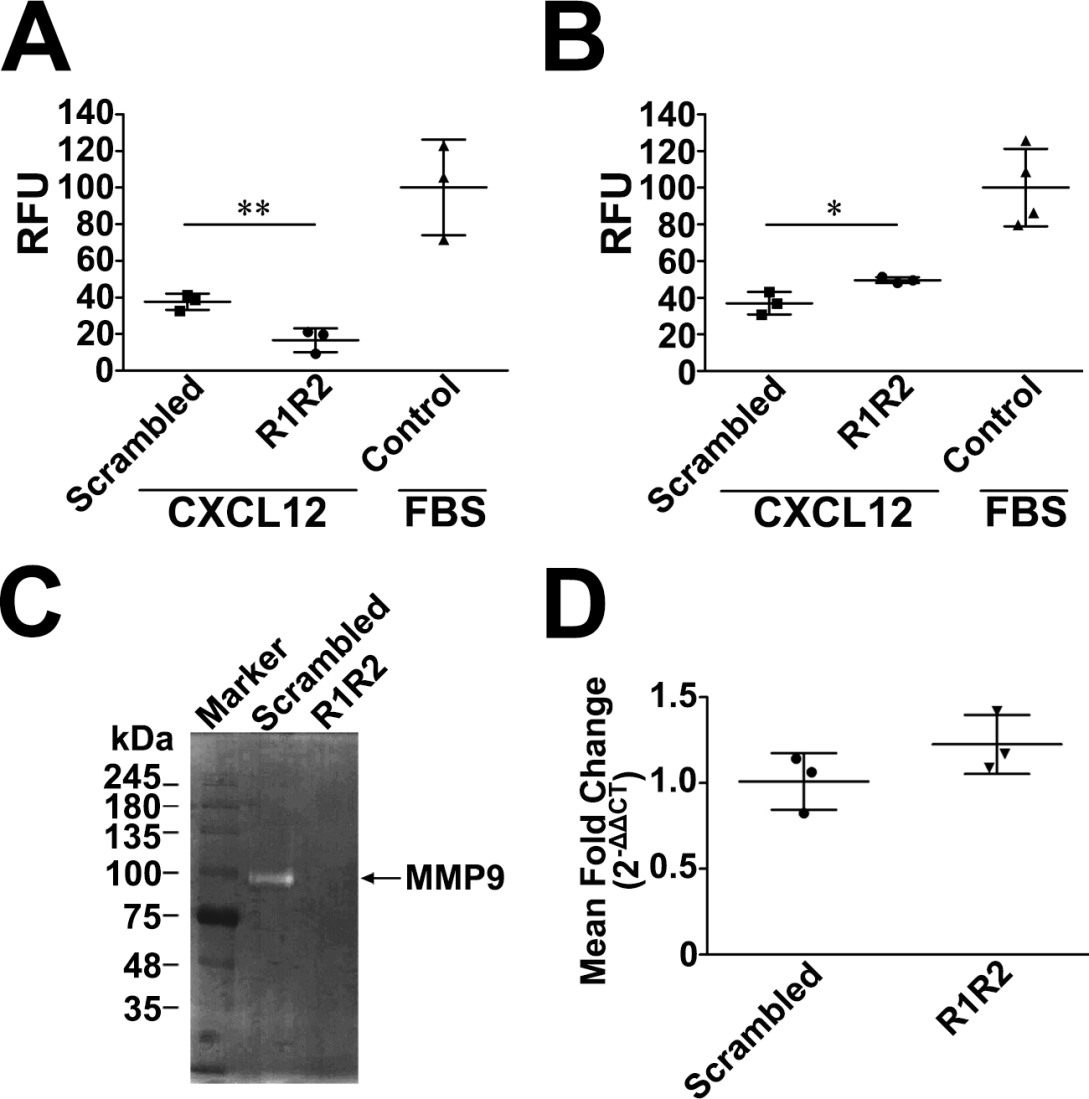
R1R2 impairs the invading capacity of fibrocytes through the BM by reducing the proteolytic activity of MMP-9. (A, B) CD45^+^, type I collagen^+^, and CXCR4^+^ fibrocytes isolated from bleomycin-treated lungs (14 days) were seeded in the upper chamber and treated with R1R2 or the scrambled peptide (1000 nM) for 24 h. CXCL12 (20 ng/mL) or 10% fetal bovine serum (FBS) were added in the bottom wells. CXCL12-induced fibrocyte invasion through Matrigel (A). Data are expressed as relative fluorescence units (RFU). The invasive capacities through Matrigel-coated inserts between R1R2- and scrambled peptide-treated fibrocytes are shown. Results were normalized for FBS-induced fibrocyte invasion. ** *P* < 0.01 by *t* test. Quantitative CXCL12-induced fibrocyte transwell migration (B). * *P* < 0.05 by *t* test. (C) Isolated fibrocytes were treated with R1R2 or scrambled peptide for 24 h. Gelatin zymography of the conditioned medium is shown. (D) MMP-9 gene expression by fibrocytes treated with R1R2 and the scrambled peptide. Gene expression was evaluated by quantitative real-time polymerase chain reaction. Data are expressed as the mean ± SD.

Our in vivo data revealed that R1R2 attenuated fibrocyte influx into the lung interstitia (Fig 5), raising the possibility that R1R2 may regulate the invasive capacity of fibrocytes to BM. To confirm the inhibitory effect of R1R2 on fibrocyte migration in vitro, we analyzed their invasive capacity by using Matrigel-coated membranes (Fig 7A). Compared with the scrambled peptide, R1R2 caused a significantly larger reduction in CXCL12-induced invasion by fibrocytes isolated from the bleomycin-treated lung (Fig 7A; 16.6 ± 6.48 relative fluorescence units [RFU, n = 3] vs. 37.58 ± 4.43 RFU [n = 3]]. Because an increase in CXCR4 expression was observed through Western blot analyses in the R1R2-treated fibrocytes (Fig 6A), we investigated whether these fibrocytes respond to their chemoattractant CXCL12 more vigorously because of the upregulated expression of CXCR4. We performed a migration assay for isolated fibrocytes through an insert without a coating. Without the obstructive Matrigel, the R1R2-treated fibrocyte migration toward CXCL12 was approximately 1.3 times higher than that in the scrambled peptide-treated fibrocytes (Fig 7B; 49.58 ± 1.63 RFU [n = 3] vs. 36.97 ± 6.15 RFU [n = 3]). Thus, R1R2 may reduce fibrocyte transmigration through BM by disabling the invasive capacity of the cells.

According to a previous study [24], migration of fibrocytes through the BM is associated with MMP-2 and MMP-9. To elucidate the mechanisms by which R1R2 inhibits fibrocyte transmigration through BM, we first evaluated the proteolytic activity of MMP-2 and MMP-9. Fibrocytes isolated from the lung digest of the bleomycin-treated mice were cultured for 48 h followed by the treatment with R1R2 or the scrambled peptide for 24 h, and the conditioned medium obtained from the fibrocytes was analyzed through gelatin zymography. Strong gelatinolytic bands corresponding to MMP-9 were observed in the fibrocytes treated with the scrambled peptide (Fig 7C). The addition of R1R2 to the cells reduced the proteolytic activity of MMP-9 induced by bleomycin treatment (Fig 7C). The proteolytic activity of MMP-2 was not detected in the scrambled peptide- or R1R2-treated fibrocytes (Fig 7C). We speculated that MMP-2 is unrelated to the inhibitory effect of R1R2 on fibrocyte invasion into fibrotic lungs. Therefore, we focused on exploring the mechanisms by which R1R2 attenuates the proteolytic activity of MMP-9 in the subsequent experiments. To elucidate whether the R1R2-induced reduction in MMP-9 activity resulted from the decrease in protein expression, we analyzed the mRNA levels of MMP-9 in isolated fibrocytes through quantitative real-time polymerase chain reaction (PCR; Fig 7D). The results revealed that adding R1R2 did not cause any difference in the transcription level of MMP-9 compared with when the scrambled peptide was added (Fig 7D; 1.23 ± 0.17 [n = 3] vs. 1.01 ± 0.17 [n = 3]).

### Binding of R1R2 to MMP-9 disables the proteolytic activity of MMP-9

**Fig 8:**
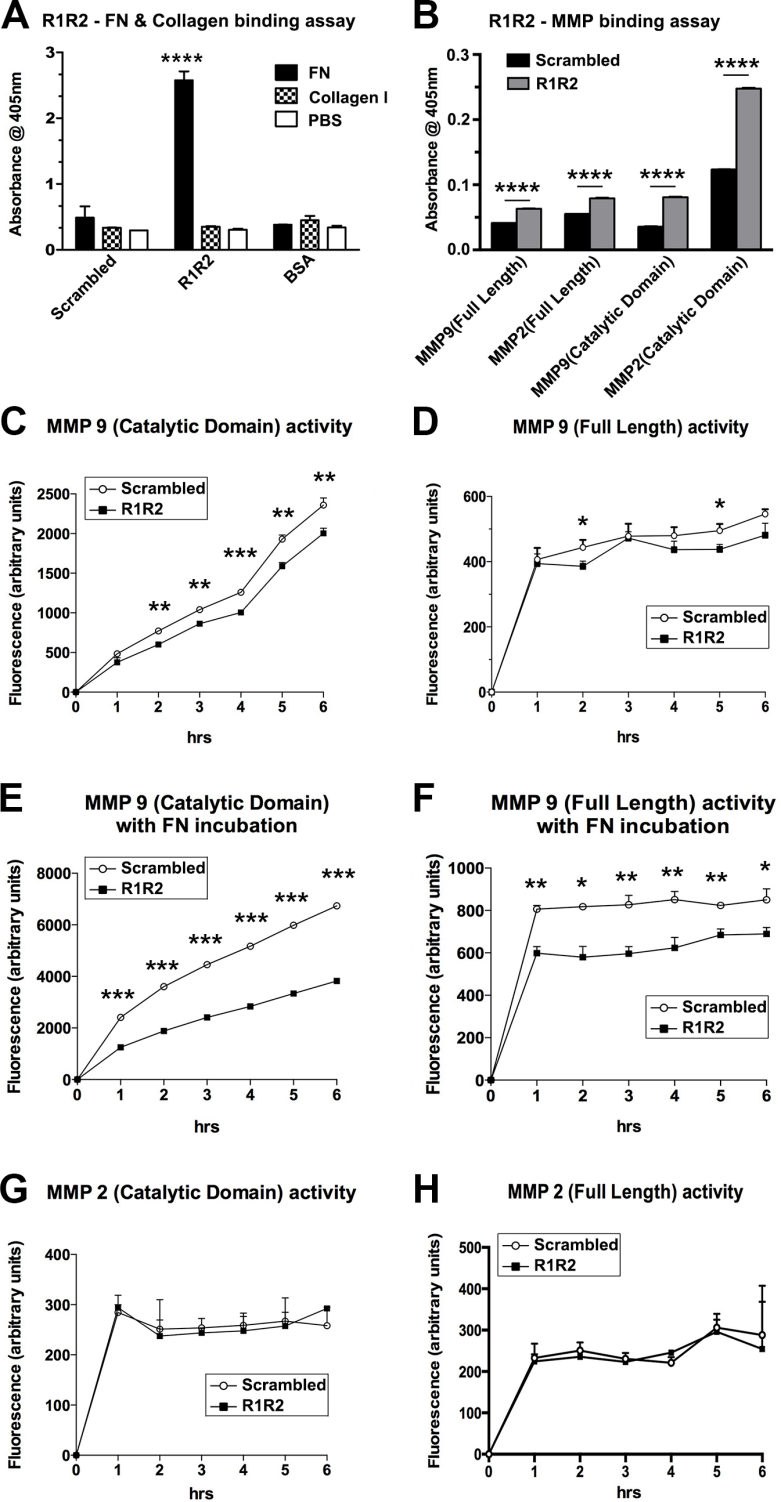
R1R2 disables the proteolytic activity of MMP-9. (A) Equal amounts of R1R2, the scrambled peptide, or bovine serum albumin (BSA; 20 μg/mL) were coated onto 96-well plates; fibronectin (FN), collagen (Col), or phosphate-buffered saline (PBS) were added into the wells, and the interaction between the peptides was evaluated by measuring the absorbance at 405 nm. **** *P* < 0.0001, one-way analysis of variance (ANOVA) followed by a Bonferroni *t* test. (B) Biotinylated R1R2 or scrambled peptide (20 μg/mL) were added into 96-well plates coated with catalytic domain or full-length proteins (FLPs) of MMP-9 and MMP-2. Bound R1R2 and the scrambled peptide were quantified at 405 nm by using a solid-phase binding assay following the incubation of anti-biotin antibodies. **** *P* < 0.0001 by t-test. (C, D) Enzymatic activity levels of catalytic domain (C) or FLPs (D) of MMP-9 were determined using a fluorescence gelatinolytic assay in the presence of 10 times the molar ratio of R1R2 or the scrambled peptide. (E, F) Enzymatic activity of MMP-9 was evaluated using a fluorescence gelatinolytic assay in the presence of 300 μg/mL (0.9 μM) FN and 20 μM R1R2 or the scrambled peptide. (G, H) Enzymatic activity of catalytic domain (G) or FLPs (H) of MMP-2 was determined using a fluorescence gelatinolytic assay in the presence of 10 times the molar ratio of R1R2 or scrambled peptide. Data are expressed as the mean ± SD. * *P* < 0.05, ** *P* < 0.01, and *** *P* < 0.001 by *t* test.

R1R2 is derived from two repeat domains of the bacterial adhesin SFS and contains a sequence similar to that of type I–IV collagen [36]. R1R2 prevents collagen–FN interactions by binding to FN [36]. Consistent with previous studies, our data revealed that R1R2 peptide binds to FN, but not to type I collagen (Fig 8A). Recent studies have reported that R1R2 binds to the gelatin-binding domain of FN, the same region that binds to the α1 chains of collagen and gelatin [37–39]. The gelatin-binding sites of FN comprise four type I and two type II FN modules. MMP-2 and MMP-9 possess a collagen binding domain (CBD), formed by three modules that resemble FN type II, inserted between the active site and the Zn^2+^-binding region in the catalytic domain [40, 41]. Because of the sequence similarity between R1R2 and collagen, raising the possibility that R1R2 competes with collagen and gelatin for binding to the CBD of MMPs, and subsequently interferes with the proteolytic activity of MMP-2 and MMP-9 on their substrates. To investigate our hypothesis, we first performed a solid-phase binding assay to determine the binding affinity of R1R2 to the catalytic domain or full-length proteins (FLPs) of MMP-2 and MMP-9 (Fig 8B). R1R2 can directly bind to the adsorbed catalytic domain or FLPs of MMP-2 and MMP-9. We subsequently characterized the functional inhibition of R1R2 in the enzymatic activity of MMPs with a fluorescent gelatin dequenching assay (Fig 8C–8H). We incubated the catalytic domain of MMP-9 with R1R2 or the scrambled peptide and observed that R1R2 was considerably more effective than the scrambled peptide in inhibiting the cleavage of fluorescence-conjugated gelatin (Fig 8C). This inhibition was also observed when R1R2 was incubated with the FLP of MMP-9 (Fig 8D). FN is an abundant soluble constituent of plasma (300 μg/mL) and a major component of ECMs [42]. Because R1R2 binds to FN with a higher affinity than it does to MMP-9, FN might reduce the R1R2 inhibition of MMP-9 activity in mice. To test this possibility, we incubated 300 μg/mL FN (normal plasma level), R1R2 or the scrambled peptide (at the concentration applied for the animals), and MMP-9 together; the results revealed that R1R2 reduced the cleavage of gelatin by MMP-9, even in the presence of FN (Fig 8E and 8F). R1R2 likely attenuated MMP-9 activity by binding to the CBD of the protease. In contrast to MMP-9, R1R2 did not regulate the proteolytic activity of MMP-2 (Fig 8G and 8H).

### R1R2 facilitates the cleavage of CXCL12 by MMP-9

**Fig 9:**
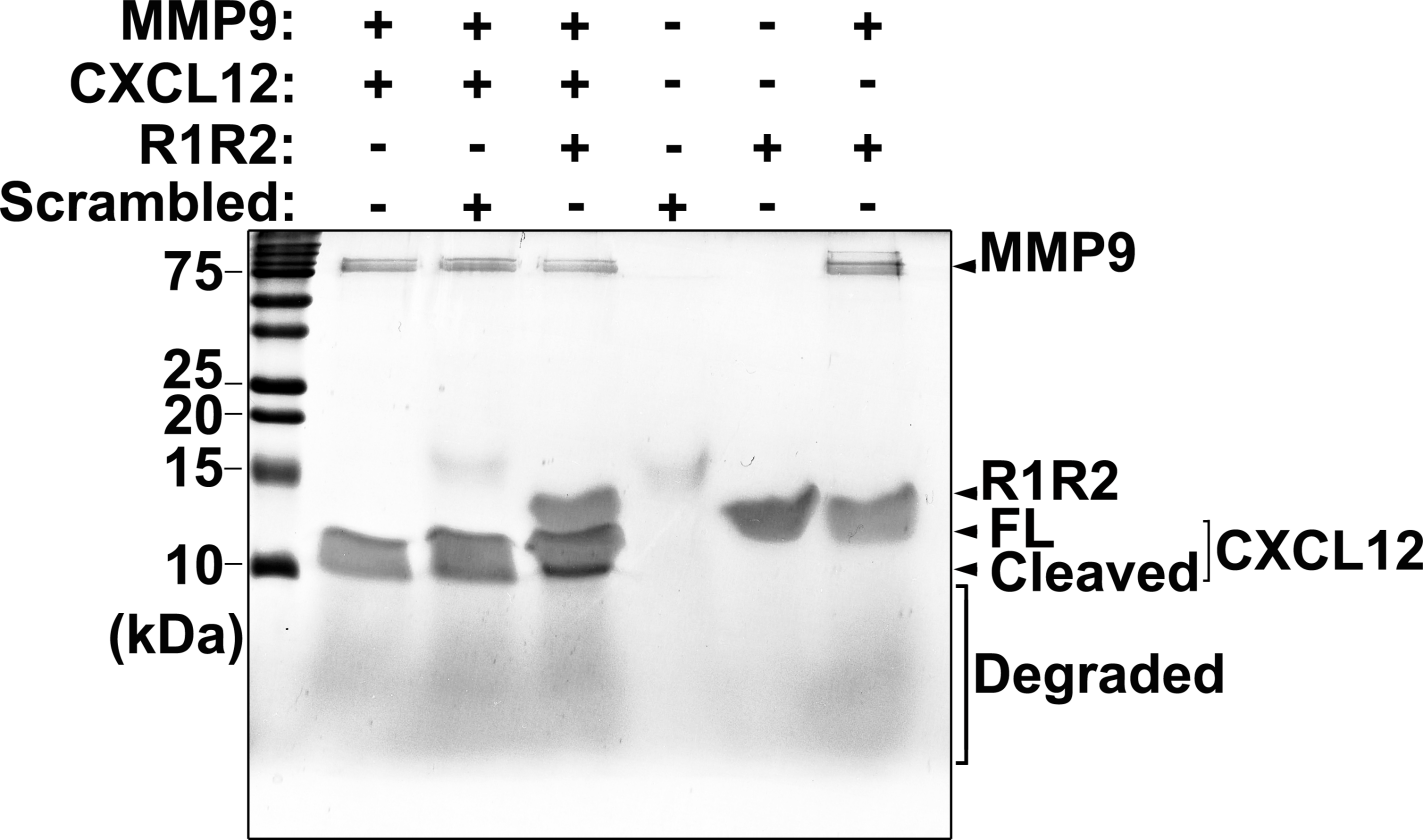
R1R2 increases CXCL12 degradation by MMP-9. CXCL12 degradation was determined by SDS-PAGE, followed by Coomassie blue staining. Lane 1: marker. Lane 2: MMP-9 (0.1 μg) was incubated with CXCL12 (2 μg) at room temperature for 3h. Lanes 3 and 4: MMP-9 was incubated with CXCL12 at room temperature for 3h with the scrambled peptide (100 μg) and R1R2 (100 μg), respectively. Lane 5 and 6: scrambled peptide and R1R2 maintained at room temperature for 3h. Lanes 7: MMP-9 was incubated with R1R2 at room temperature for 3h. The smear at the bottom of the gel indicated the slightly degraded products of proteins.

The CXCR4/CXCL12 chemokine axis is critical in trafficking and homing circulating fibrocytes [19]. The functional blockade of CXCL12 with antibodies inhibits fibrocyte migration and differentiation to SMAA^+^ fibroblasts or myofibroblasts [43]. Therefore, the methods for promoting CXCL12 degradation have therapeutic potential for pulmonary fibrosis alleviation. CXCL12 is cleaved by MMP-9, causing its receptor CXCR4 to lose its binding ability [44, 45]. Our results revealed that R1R2 inhibited the proteolytic activity of MMP-9 on gelatin (Fig 8). To determine whether R1R2 regulates the MMP-9-mediated CXCL12 cleavage, the mixtures of full-length MMP-9 and CXCL12 were incubated with R1R2 or the scrambled peptide at room temperature (27°C) for 3 h. The incubation of MMP-9 with CXCL12 induced CXCL12 degradation, as indicated by a faint cleaved fragment observed below CXCL12 (Fig 9 and S2 Fig). The addition of R1R2 to the mixtures of MMP-9 and CXCL12 promoted the production of a robustly cleaved fragment below CXCL12. By contrast, the addition of the scrambled peptide to the mixtures did not induce more CXCL12 degradation. Thus, the binding of R1R2 to MMP-9 reduced the cleavage of gelatin and simultaneously increased the degradation of CXCL12.

## Discussion

In this study, we demonstrated that R1R2 is an effective inhibitor of bleomycin-induced pulmonary fibrosis. Our in vivo data reveal that intratracheal delivery of R1R2 prevented collagen deposition and considerably decreased the presence of SMAA^+^ myofibroblasts in the parenchyma of the fibrotic lung (Figs 1–3). Moreover, R1R2 significantly reduced bleomycin-induced fibrocyte recruitment by flow cytometry analysis and myofibroblast differentiation using Western blotting and double immunofluorescence of SMAA and CXCR4 (Figs 4–6). Additionally, R1R2 blocked fibrocyte invasion through BM-like proteins in vitro (Fig 7). Furthermore, the binding of R1R2 to MMP-9 reduced the cleavage of gelatin (Fig 8) and simultaneously promoted the degradation of CXCL12 by MMP-9 (Fig 9).

Myofibroblasts produce excess deposition of a collagen-rich ECM in the interstitia of the lungs, leading to irreversible damage to the lung architecture and lung fibrosis [1, 2]. Studies have reported multiple potential sources of lung fibroblasts or myofibroblasts, including resident mesenchymal cells, pulmonary epithelial cells in epithelial–mesenchymal transitions, and fibrocytes [9, 10]. Fibrocytes are derived from the circulating mesenchymal progenitor cells [11–14] and proliferate rapidly and leave the bloodstream to enter sites of injury upon pathological stimulation [15, 16]. In our study, we performed double immunofluorescence of CXCR4 and SMAA in lung sections of the bleomycin-treated mice for 14 days (Fig 4I–4N), indicating that R1R2 treatment apparently decreased the cells double positive for CXCR4 and SMAA compared with the scrambled peptide treatment. The CXCR4 ^+^ cells co-express SMAA in the fibrotic lungs, indicating that these CXCR4-expressing cells were directly differentiating into myofibroblasts. Conceptually, we delivered R1R2 into the mice the next day after bleomycin administration and continued the treatment every other day till harvest of the lungs. Immediate therapy, often termed ‘preventive’, is the least relevant to human pulmonary fibrosis. Timing therapy to the most relevant mechanistic time point would be needed in the future in order to investigate whether R1R2 treatment in the later stage of pulmonary fibrosis will inhibits the differentiation of fibrocytes into myofibroblasts in the fibrotic lungs. Although we haven’t done this experiment, however, based on our in vitro results that R1R2 attenuates fibrocyte differentiation (Fig 6), we anticipate that R1R2 can prevent further deterioration of fibrosis by blocking fibrocyte differentiation into myofibroblasts.

In our previous study, we reported that R1R2 functions as a potent inhibitor, reducing type I collagen deposition in injured vessels [26]. In addition to directly binding on FN to block excess ECM collagen deposition, our present study highlights a novel function of R1R2—preventing the fibrocyte influx into the lung interstitial in the pathogenesis of pulmonary fibrosis and inhibiting fibrocyte differentiation into myofibroblasts. Our data show that R1R2 reduces the trafficking and homing of fibrocytes in two routes: (1) R1R2 disables the activity of MMP-9 toward the BM, thus lowering the invasion capacity of fibrocytes. (2) R1R2 promotes the cleavage of the fibrocyte chemoattractant CXCL12, thus disrupting the CXCR4/CXCL12 axis[19].

Our quantitative real-time PCR results reveal that R1R2 does not regulate the function of MMP-9 by altering its biosynthesis (Fig 7D). We confirmed that R1R2 inactivates MMP-9 by binding to the catalytic domain of the protease (Fig 8). The general structure of MMPs contains a predomain (which is cleaved during enzyme activation), propeptide, catalytic domain, and carboxyterminal hemopexin-like domain [46]. MMP-9 and MMP-2 possess a unique CBD, comprising three FN type II repeats inserted into the catalytic domain [40, 41, 47, 48]. In addition to the native and denatured type I collagen, the CBD of MMP-9 and MMP-2 binds to elastin, type IV and V collagen, as well as heparin [49]. The CBD of MMP-9 aids in positioning the substrates for cleavage, and it is required for the hydrolysis of type IV collagen and gelatin, but not that of short peptides [40, 50, 51]. R1R2 contains a sequence homologous to type I–IV collagen [36]. A recent study located the site where R1R2 binds to the gelatin-binding domain of FN, preferentially at the eighth or ninth FN type I module [37]. To date, although there is no evidence showing that R1R2 can bind to FN type II modules, FN type I modules have a 42% sequence similarity and 28% sequence identity with the type II modules [51]. The solid-phase binding assay indicated lower binding affinity of R1R2 with MMP-9 compared with the interaction between R1R2 and FN (data not shown). Nonetheless, even the binding affinity of R1R2 with MMP-9 was low; R1R2 did defect the proteolytic activity of MMP-9 on gelatin (Fig 8). We speculate that the unstable interaction between R1R2 and MMP-9 may cause conformational changes in MMP-9, interfering with the binding of gelatin and CBD and may further impair the subsequent cleavage of gelatin by MMP-9.

Our research results indicate that the inhibitory effect of R1R2 occurs only to MMP-9, but not to MMP-2 (Fig 8). MMP-9 and MMP-2 are gelatinases; they share many characteristics, such as their primary structures and substrate specificities [52]. Otherwise, MMP-9 contains a unique 54 amino acid-long and proline-rich module in the catalytic domain. This proline-rich region is homologous to the α2 chain of collagen type V and spans the zinc binding and hemopexin domains [51, 53, 54]. The function of this proline-rich domain remains unclear; the function has been hypothesized to be the formation of multimeric complexes with other gelatinases and ECM proteins or to function as an additional site for macromolecular substrate recognition [51, 53]. The proline-rich domain may function in a manner that facilitates the inhibition of the proteolytic activity of MMP-9 by R1R2. Moreover, the CBD of MMP-2 regulates the proteolytic activity of MMP-9 on its substrates [41]. Therefore, R1R2 binding to MMP-2 may indirectly reduce gelatin degradation by its modulation of MMP-9 activity. In addition to reducing the activity of MMP-9 on gelatin, our data reveal that R1R2 promotes the proteolytic activity of MMP-9 on CXCL12, thus disrupting the CXCR4/CXCL12 axis (Fig 9). These data indicate that R1R2 has dual simultaneous regulatory effects on MMP-9—reducing gelatin cleavage and increasing CXCL12 degradation. Thus, R1R2 may be a unique inhibitor of MMP-9 because low molecular weight inhibitors may bind to the active sites of MMPs and typically block a wide range of MMP activities on substrates [51]. However, R1R2 also functions as an enhancer to promote the proteolytic activity of MMP-9 on CXCL12.

Our in vivo and in vitro data reveal that R1R2 reduces SMAA expression in fibrotic lungs and isolated fibrocytes (Figs 3, 4 and 6), implying that R1R2 prevents fibrocyte differentiation to myofibroblasts. TGF-β, which is well-characterized regarding its role in regulating fibrocyte differentiation [16, 55], is secreted as an inactive complex with a latency-associated peptide (LAP). This complex interacts with latent TGF-β binding proteins (LTBPs) and is anchored in the ECM [56, 57]. TGF-β is liberated from this complex through TGF-β activation [57, 58]. TGF-β may be released by the proteolytic cleavage of LAP, LTBP, or ECM proteins with several proteases including MMP-9 [57–59]. In the present study, R1R2 reduced the proteolytic activity of MMP-9 (Fig 8), suggesting that R1R2 prevents fibrocyte differentiation to myofibroblasts by blocking MMP-9 mediated TGF-β activation. Another possible mechanism by which R1R2 may prevent fibrocyte differentiation is by increasing CXCL12 degradation by MMP-9 (Fig 9). The CXCR4/ CXCL12 axis contributes to pulmonary fibrosis development [19, 60]. In addition to functioning as a chemoattractant to recruit fibrocytes into the fibrotic lungs [19], CXCL12 alone can facilitate fibrocyte differentiation myofibroblasts by ligating to its receptor CXCR4 [61]. We postulate that R1R2-mediated MMP-9 cleavage on CXCL12 results in the loss of binding between CXCL12 and CXCR4, inhibiting fibrocyte differentiation.

FN, a multidomain glycoprotein, is secreted as a dimer maintained by two disulfide bonds [38, 42, 62]. R1R2 is a recombinant peptide containing two copies of collagen-like 19-residue repeats separated by a linker peptide [25, 26, 36]. Each repeat has a conserved motif, similar to the sequences on α1 sequences from type I collagen; this motif binds to the type I module of a single FN monomer [25, 26, 36]. FN is abundantly distributed in the plasma and forms the major component of ECMs [42]. A recent study demonstrated that R1R2 bound to FN subunits forms the stable complex [37]. Although our data reveal that R1R2 reduced the cleavage of gelatin by MMP-9, even in the presence of FN (Fig 8), R1R2 administered to animals will still bind to the plasma FN, which will decrease the efficacy of the peptide. Therefore, in the future, modification of R1R2 peptide, which can bind to MMP-9, but not to FN, may provide a superior therapeutic option for attenuating pulmonary fibrosis.

In summary, our experimental results provide strong evidence indicating that binding of R1R2 on MMPs has dual regulation on MMP-9 through reduced enzymatic activity on gelatin and increased cleavage of CXCL12. Through its regulation on MMP-9, R1R2 inhibits pulmonary fibrosis by abolishing fibrocyte infiltration across the subendothelial BM into the parenchyma of the lungs and decreasing fibrocyte differentiation into myofibroblasts. These data highlight the therapeutic potential of R1R2 in treating pulmonary fibrosis in the future.

## Supporting information

S1 Fig Dose-dependent effects of bleomycin-induced pulmonary fibrosis. Collagen content was biochemically measured using a hydroxyproline assay in the lung 14 days after bleomycin administration. ** *P* < 0.01 by Kruskal–Wallis test, followed by Dunn’s multiple comparisons test.
(TIF)

S2 Fig R1R2 increases CXCL12 degradation by MMP-9. CXCL12 degradation was determined by SDS-PAGE, followed by Coomassie blue staining. Lane 1: marker. Lane 2: MMP-9 (0.1 μg) was incubated with CXCL12 (2 μg) at room temperature for 3h. Lanes 3 and 4: MMP-9 was incubated with CXCL12 at room temperature for 3h with the scrambled peptide (100 μg) and R1R2 (100 μg), respectively. Lane 5 and 6: scrambled peptide and R1R2 maintained at room temperature for 3h.
(TIF)
