# Supplementary figures and images for: R1R2 peptide ameliorates pulmonary fibrosis in mice through fibrocyte migration and differentiation
Source: PLoS One. 2017 Oct 2;12(10):e0185811. doi: 10.1371/journal.pone.0185811 (PMC5624629; doi:10.1371/journal.pone.0185811)

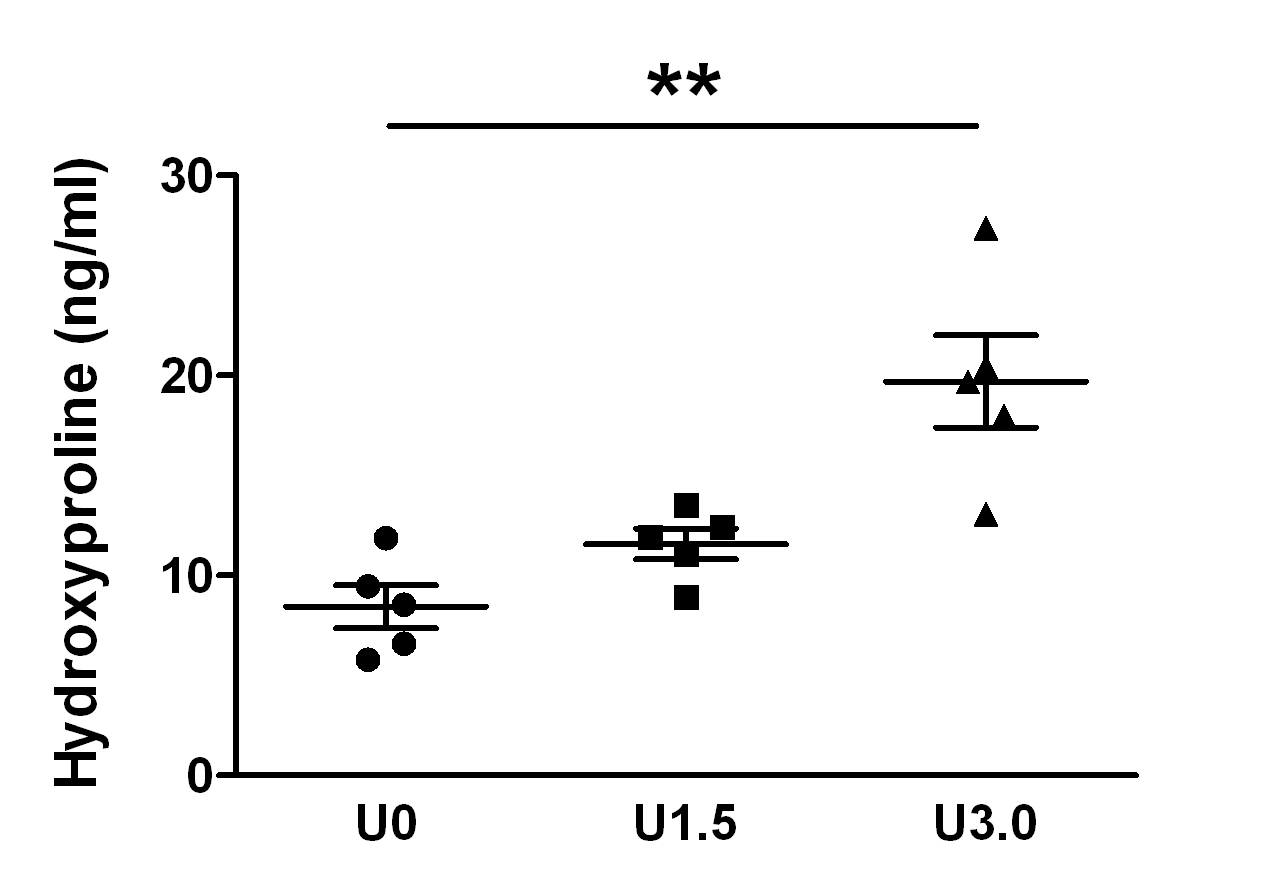

Supplement: S1 Fig — Collagen content was biochemically measured using a hydroxyproline assay in the lung 14 days after bleomycin administration. ** P < 0.01 by Kruskal–Wallis test, followed by Dunn’s multiple comparisons test. (TIF) [file pone.0185811.s001.tif]

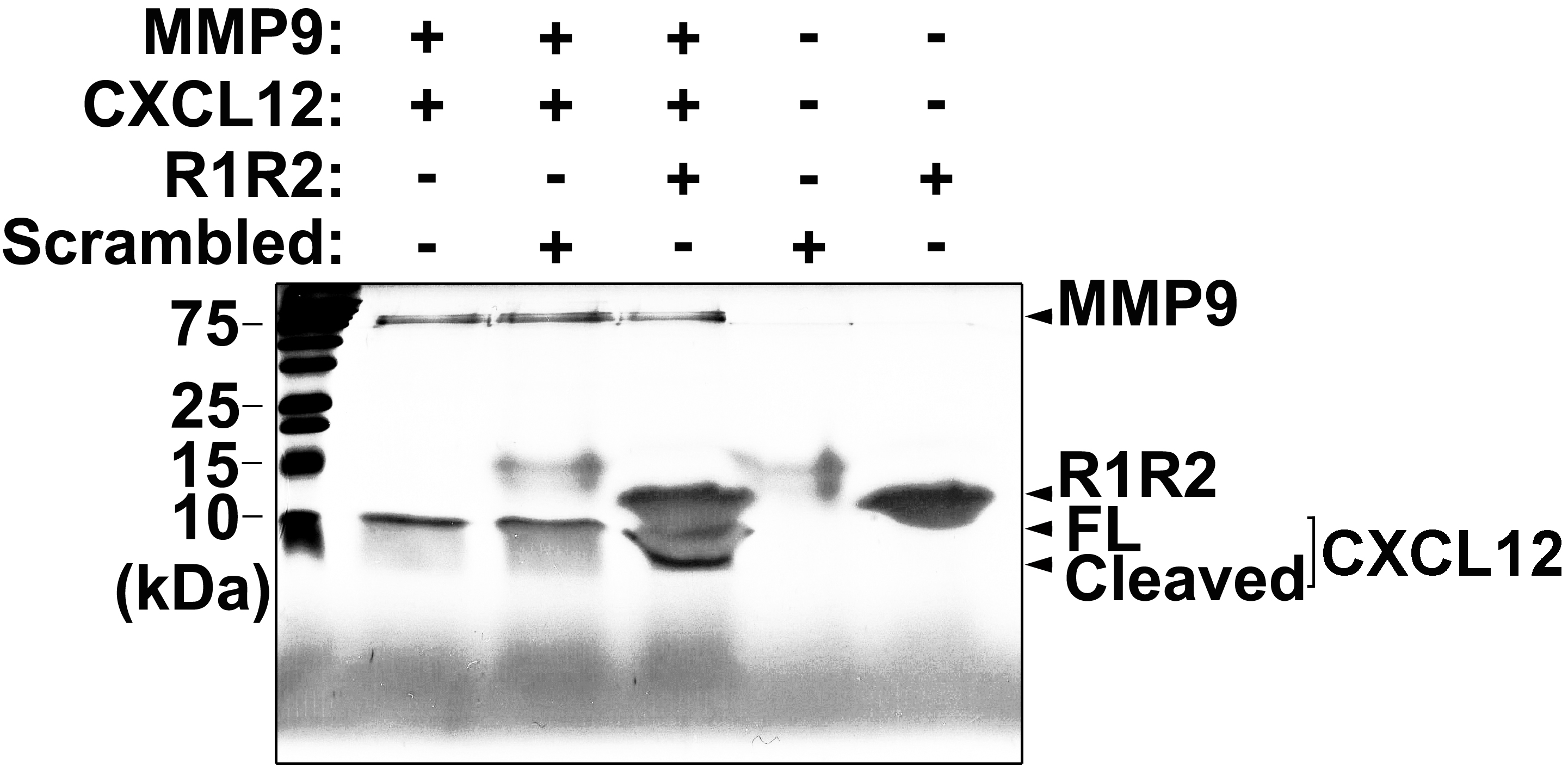

Supplement: S2 Fig — CXCL12 degradation was determined by SDS-PAGE, followed by Coomassie blue staining. Lane 1: marker. Lane 2: MMP-9 (0.1 μg) was incubated with CXCL12 (2 μg) at room temperature for 3h. Lanes 3 and 4: MMP-9 was incubated with CXCL12 at room temperature for 3h with the scrambled peptide (100 μg) and R1R2 (100 μg), respectively. Lane 5 and 6: scrambled peptide and R1R2 maintained at room temperature for 3h. (TIF) [file pone.0185811.s002.tif]
